# Supplementary figures and images for: RNA-Sequencing of Tumor-Educated Platelets, a Novel Biomarker for Blood-Based Sarcoma Diagnostics
Source: Cancers (Basel). 2020 May 27;12(6):1372. doi: 10.3390/cancers12061372 (PMC7352477; doi:10.3390/cancers12061372)

Figure S1

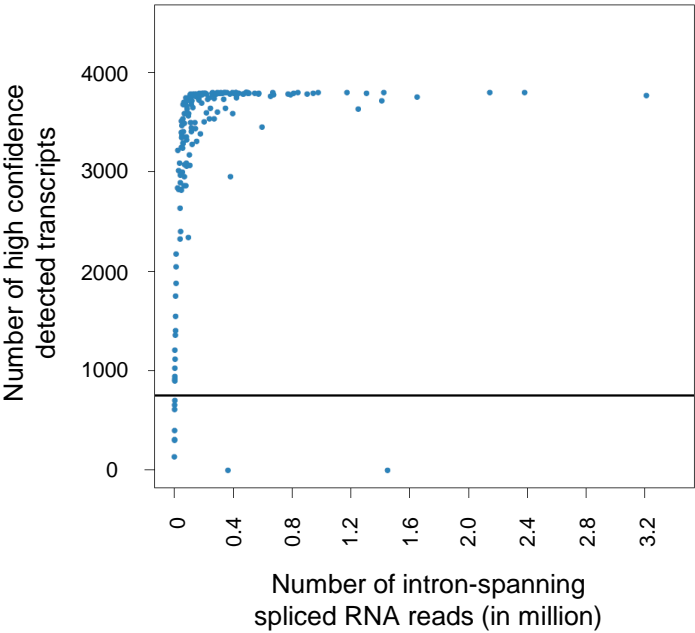

(a) Confidence plot

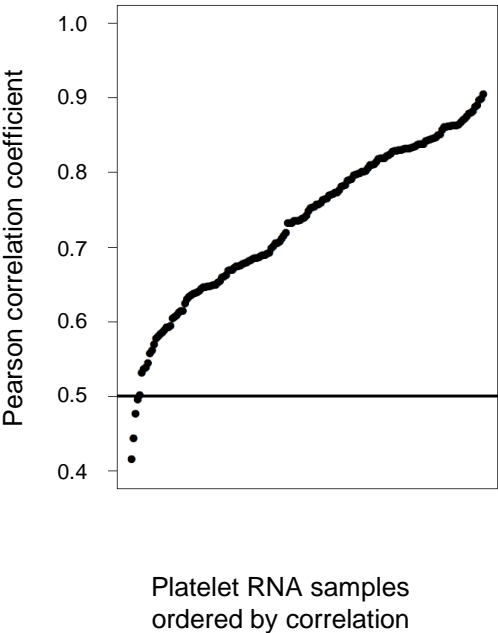

(b) Correlation analysis

Figure S2

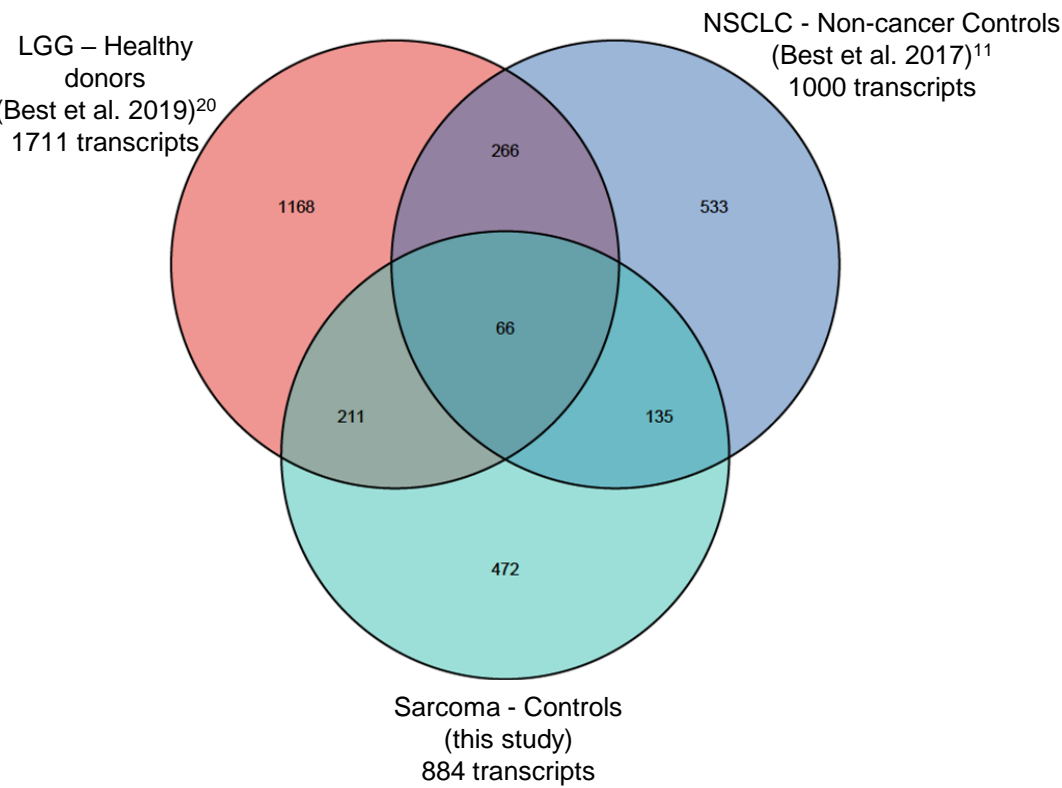

Supplement: Supplementary file 1 [file cancers-12-01372-s001.zip › Supplementary Figures.pdf]
